# Effect of Traditional Chinese Medicine Product, QiangGuYin, on Bone Mineral Density and Bone Turnover in Chinese Postmenopausal Osteoporosis

**DOI:** 10.1155/2017/6062707

**Published:** 2017-04-20

**Authors:** Zhen-Yu Shi, Xin-Gen Zhang, Chun-Wen Li, Kang Liu, Bo-Cheng Liang, Xiao-Lin Shi

**Affiliations:** ^1^The Second Clinical Medical College, Zhejiang Chinese Medical University, Hangzhou, Zhejiang 310053, China; ^2^Department of Orthopedics and Traumatology, Rongjun Hospital of Zhejiang, Jiaxing, Zhejiang 314001, China; ^3^Department of Diagnostics of Traditional Chinese Medicine, College of Basic Medical Science, Zhejiang Chinese Medical University, Hangzhou, Zhejiang 310053, China; ^4^Department of Orthopedics and Traumatology, The Second Affiliated Hospital of Zhejiang Chinese Medical University, Zhejiang 310005, China

## Abstract

*Introduction*. The aim of this study was to investigate the efficacy of herbal formula QiangGuYin (QGY) in postmenopausal women.* Materials and Methods*. A total of 240 participants from six clinical centers were randomly to receive alendronate 70 mg/week, QGY granules 20 g/day, and placebo. Primary end points were BMD changes over 6 and 12 months; secondary end points were bone turnover markers changes at 3, 6, 9, and 12 months. Safety was monitored by clinical adverse events reported during the follow-up.* Results*. Of 240 women recruited, 218 completed the study. Significant BMD increases from baseline were observed over 6 and 12 months at each observed part both in QGY and alendronate compared with placebo (*p* < 0.01). Alendronate-treated subjects had significant decreases in *β*-CTX compared to QGY-treated subjects at each time point assessed (*p* < 0.01). Reduction in t-P1NP was only observed in the QGY group at 3 and 6 months (−23.81% and −3.07%, resp.). No significant difference was observed in the overall incidence of clinical adverse events among the alendronate group and the QGY group (5.0% versus 7.5%, *p* = 0.513).* Conclusion*. 1-Year treatment with QGY demonstrated a safe statistical increase in BMD and new balance may be rebuilt after 9 months. This trail is registered with ChiCTR-POC-16008026.

## 1. Introduction

Osteoporosis, a global public health problem, is considered to be a major health issue secondary to coronary heart disease by World Health Organization (WHO) [1]. In China, it is estimated that over 90 million people are suffering from osteoporosis [2]. Despite the high prevalence and potentially devastating impact of osteoporosis, these patients are usually diagnosed till a fragility fracture occurs.

Major therapeutic options including bisphosphonates, calcitonin, estrogen, Vitamin D analogs, selective estrogen receptor modulators (e.g., raloxifene and droloxifen), and recombinant human parathyroid hormone (PTH) 1–34 (teriparatide) are available to postmenopausal women for prevention and treatment of osteoporosis. The efficacy mechanisms, both the reversible inhibition in osteoclast-mediated bone resorption and the moderate acceleration in osteoblast-mediated bone formation, are well documented [3]. Unfortunately, many patients are still not willing to be treated in daily life and show impatience and noncooperation during the course of therapy. Reasons for this in seniors may be a perceived lack of evidence that these patients benefit from these treatments [4] and are unduly concerned about the adverse reactions. In the meantime, pharmacologic treatment is discussed by specialists and scholars on the topic of the benefits and rare potential risks [5].

Traditional Chinese medicine (TCM) gradually attracts the attention. According to Chinese special historical cultural environment and social background, TCM have long been widely used in clinical practice to prevent and treat osteoporosis and many other bone diseases. Due to the fewer side effects and being more suitable for long-term application compared with other chemically synthesized medicines, TCM have received extensive attention. TCM theories have extensive experience accumulated over thousands of years [6]. The therapeutic effect of TCM enjoys popular support. More and more researchers from home and abroad try to verify the pharmacological mechanisms, but Chinese physicians prescribe one or more herbal formulae combined with several single herbs in each prescription to match different patient's constitution [7], which also brings numerous uncertainties and difficulty in modern scientific research.

Postmenopausal osteoporosis (type I osteoporosis) is the most common disease in women after menopause, which is linked to an estrogen deficiency. Based on over ten years of clinical practice, the guideline that TCM with qi-tonifying and meridian-warming effects have the potential effects on treating osteoporosis is summarized from experience [8]. QiangGuYin is based on the combination therapy of Chinese medicine for tonifying qi and warming meridians. The main objective of the present study was to assess the effect of traditional Chinese medicine product, QiangGuYin, on bone mineral density and bone markers among postmenopausal women with osteoporosis.

## 2. Methods

### 2.1. Study Design

This 12-month, multicenter, randomized, open-label, placebo-controlled study was conducted in Xinhua Hospital, Zhongshan Hospital, Rongjun Hospital, Bo'ai Hospital, Haiyan Hospital of Zhejiang Province, and the Second Affiliated Hospital of Heilongjiang University of Chinese Medicine from March 2013 to May 2015. After screening between September 2013 and March 2014, 240 participants were randomly assigned in a 1 : 1 ratio to receive either alendronate 70 mg/week (alendronate group, Merck Sharp & Dohme (Italia) S.p.A., J20130085), TCM prescription QiangGuYin granules 20 g/day (QGY group, concentrated decoction, composed of Cornu Cervi Degelatinatum 20 g, honeysuckle stem 25 g, Caulis Spatholobi 25 g,* Gentiana macrophylla* 15 g, Radix Sileris 15 g, Nidus Vespae 20 g, cinnamon 10 g,* Ligusticum wallichii* 20 g,* Astragalus membranaceus* 30 g, Rhizoma Drynariae 20 g,* Eucommia ulmoides* 15 g, and* Dipsacus asperoides* 30 g, produced by Pharmaceutical Preparation Centre of the Second Affiliated Hospital of Heilongjiang University Of Chinese Medicine), or placebo (placebo group). Randomization was performed according to computer-generated randomization list and stratified to each hospital provided by the study center. All patients were instructed on the correct way to take the tablets, as per the manufacturer's doing instructions. Drug distribution was performed at each clinical center every 3 months. To be taking 800 mg of calcium daily in food and giving informed consent to participate are required in the study.

### 2.2. Patients

Eligible patients were generally healthy women with 45 to 70 years of age who were at least 1 year postmenopausal with a bone mineral density *T*-score of less than −2.5 at the lumbar spine or the superior hip or with a history of osteoporotic fractures.

Exclusion criteria included selected cancers (e.g., breast), secondary causes of osteoporosis (e.g., Cushing's disease, hyperthyroidism, Crohn's disease, or rheumatoid arthritis), hypercalcemia or hypocalcemia, serious cardiovascular disease, or kidney failure. Women who were treated with drugs that were potentially able to alter bone metabolism or switched more than one osteoporosis drug differing from their initial therapy were excluded from the trial. Use of oral bisphosphonates, parathyroid hormone, sodium fluoride, strontium ranelate, calcitonin, testosterone, systemic glucocorticoids, or anabolic steroids and any investigational therapy except the study medication was prohibited throughout the trial [9].

### 2.3. End Points

The primary end point was percent change from baseline in bone mineral density at the lumbar spine, total superior hip, femoral neck, and hip trochanter at 6 and 12 months. An increase of 1% in QGY group was the excepted response to express its efficacy. Secondary end points were changes in bone turnover markers (BTM) of total procollagen type 1 aminoterminal propeptide (t-P1NP) and the serum *β*-isomerized C-terminal telopeptide of type 1 collagen (*β*-CTX) during the same trial period [10, 11].

### 2.4. Adverse Events

Every 3 months after each study drug administration, patients were evaluated for the following trial including vital signs, body weight, laboratory tests (hematologic and chemical measurements and urinalysis), and physical examinations and questioned for the occurrence of adverse events. Clinically relevant changes and possible spontaneous adverse events were documented as adverse or severe adverse events.

### 2.5. Study Measurements

BMD of the lumbar spine (L1–L4), total hip, trochanter, and femoral neck in the anteroposterior view was measured by dual-energy X-ray absorptiometry (DXA) using Osteocore 2 (MEDILINK, France) at baseline and months 6 and 12. Instruments' quality control includes standard deviation (0.0032), variability range (0.41%), age grouping (15–95 years old), weight (<150 kg), and bone mineral density range (0.3–1.4, +/−1.0% in vivo).

For bone turnover markers, serum samples were collected at approximately the same time in the morning after an overnight fast and delivered to the central laboratory (DIAN Diagnostics, Hangzhou, China) for measurement at baseline and 3, 6, 9, and 12 months. Bone turnover markers (*β*-CTX and t-P1NP) levels were measured by electrochemiluminescence immunoassay (Cobas e601 automated immunoassay analyzer, Roche, Germany) using Roche commercial kits.

### 2.6. Statistical Analysis

Sample size (*n*) in each group was computed as [8, 12]

(1) n=2Uα+UβSδ2,Sδ=CVpercentage  difference.

The trial was designed to give 1 − *β* = 90% power (*β* = 10%; *U*_*β*_ = 1.28) to detect a 1% difference from baseline with respect to change in BMD over 12 months, assuming a measurement precision error of 1.7% and setting *α* at 0.05 (*U*_*α*_ = 1.96). Allowing for a drop-out rate of 15%, a sample of *n* = 80 in each group was therefore planned to be randomized.

Data are expressed as means ± standard deviation (SD) or standard error (SE). Baseline date was verified by one-way analysis of variance (ANOVA) with repeated measurements. The significance of percentage changes comparisons between the chosen double groups was determined by the unpaired *t*-test. All statistical analyses were performed using the SPSS 19.0 program. A significance level of *p* < 0.05 was used for all comparisons. Primary efficacy results were analyzed in the modified intention-to-treat population (participants who underwent baseline and one or more postbaseline assessments of the primary efficacy variable). In the case of missing data on BTM and BMD at 12 months, the data were input with the use of the last-observation-carried-forward method. If a month 9 BTM was missing, it was assumed that there was no percent change at month 6. Incidence between the QGY and alendronate groups with regard to safety was descriptive and unadjusted for multiple comparisons; *p* values were based on Pearson's Chi-Square.

## 3. Result

### 3.1. Study Disposition

**Figure 1:**
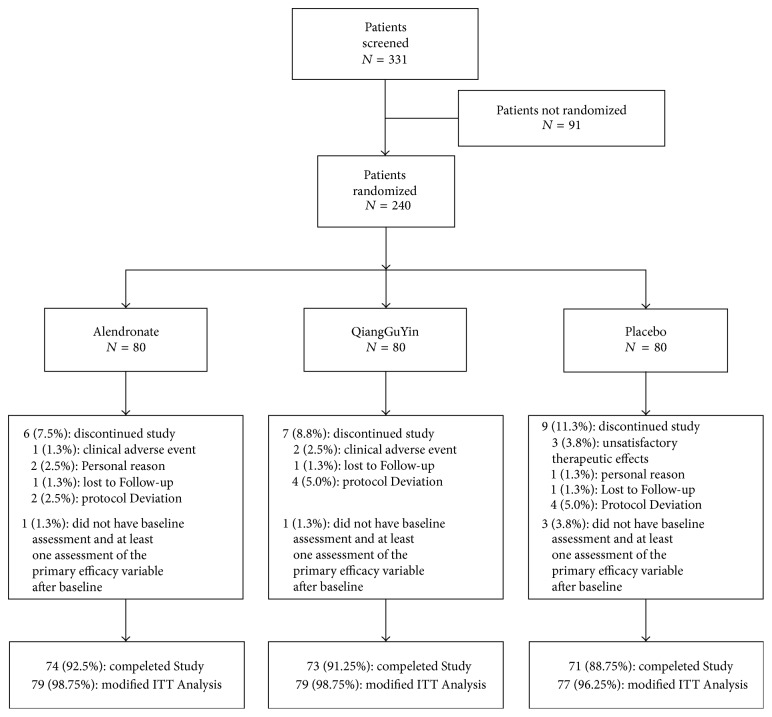
Subject disposition. Flow diagram of the phases of the randomized trial.

In total, 331 patients were screened at six study sites in China and a total of 240 patients (80 alendronate, 80 QiangGuYin, and 80 placebo) received at least one stage of this study. After MITT (modified intention-to-treat), the completion rates for this 12-month trial in the three treatment groups (alendronate, 98.75%; QGY, 98.75%; Placebo, 96.25%) were similar (Figure 1).

Baseline demographics and characteristics were balanced between the treatment groups. Patients' baseline information, BMD, and biochemical marker values are presented in Table 1.

### 3.2. Efficacy

**Figure 2:**
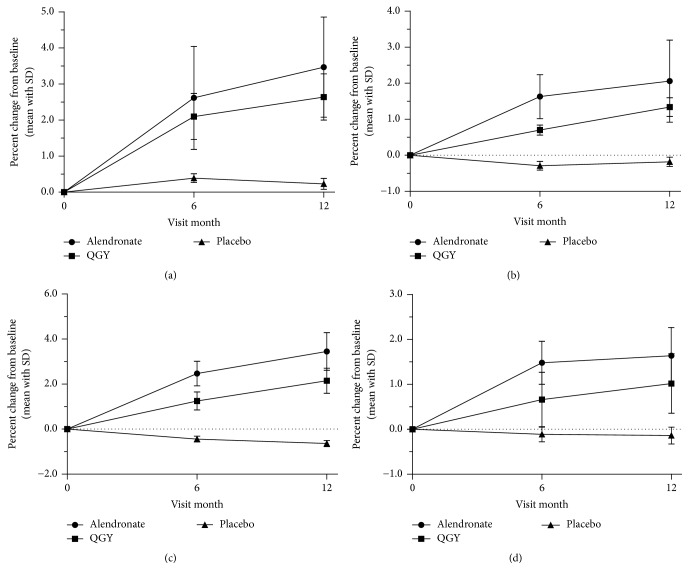
Mean percent changes in the bone mineral density of (a) lumbar spine BMD, (b) total hip BMD, (c) hip trochanter BMD, and (d) femoral neck BMD from baseline to month 12. Data are presented as the mean ± standard deviation.

For primary end point, regarding BMD, significant increases from baseline were observed at 6 and 12 months both in QGY and alendronate compared with placebo (*p* < 0.01, Figure 2). At 12 months, the BMD significantly increased from the baseline level in alendronate group (+3.47%, +2.06%, +3.45%, and +1.64% in lumbar spine BMD, total hip BMD, hip trochanter BMD, and femoral neck BMD, resp.) and remained higher than the QGY group level (+2.64%, +1.34%, +2.15%, and +1.02%, resp.; Figure 2).

The standard difference (SD) at 12 months in alendronate was greater than QGY except in femoral neck (0.63 versus 0.66, Figure 2(d)). More rapid gains in BMD were seen with alendronate than with QGY in first 6 months but not throughout this trial: the slope of line segments corresponding to the date rangeability from 6 months to 12 months between 2 groups in total hip (0.071 versus 0.106, Figure 2(b)) and femoral neck (0.026 versus 0.060, Figure 2(d)).

**Figure 3:**
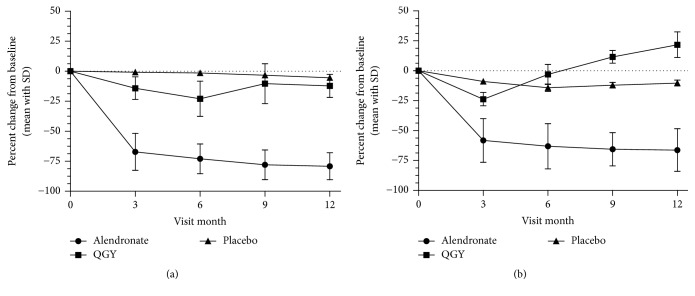
Percent changes of (a) serum *β*-isomerized C-terminal telopeptide of type 1 collagen (*β*-CTX) and (b) total procollagen type 1 aminoterminal propeptide (t-P1NP) in months 3, 6, 9, and 12 compared with the baseline. Data are presented as the mean ± standard deviation.

For secondary efficacy end points, regarding bone turnover markers, biochemical markers of bone turnover were reduced in both the QGY and alendronate groups (Figure 3). In QGY-treated subjects, *β*-CTX reduction was maintained, with maximal mean decreases from baseline observed at month 6 (−22.97%; Figure 3(a)), but was significantly less than that observed for alendronate-treated subjects (−72.97%; *p* < 0.01). Similarly, at month 3, mean decreases were less in the QGY group than in the alendronate group (−14.16% versus −67.18%, resp.; *p* < 0.01). At month 9, subjects received the second dose of QGY; an increase in *β*-CTX was observed compared with month 6 (−10.29%, month 9, and −22.97%, month 6; *p* < 0.01). At month 12, the mean decreases in *β*-CTX were similar for both neighboring point data (−12.22%, month 12, versus −10.29%, month 9; *p* = 0.374) but were still significantly less than that noted in the placebo-treated subjects (−5.38%; *p* < 0.01). Alendronate-treated subjects had significantly greater decreases in serum concentrations of *β*-CTX than QGY-treated subjects at each time point assessed (*p* < 0.01; Figure 3(a)).

A rapid fall in the bone formation marker t-P1NP also was observed for both treatment groups during the first 3 months. At month 3, t-P1NP levels decreased from baseline to −23.81% in the QGY group and −58.14% in the alendronate group (*p* < 0.01); reduction in t-P1NP was only observed in the QGY group by month 3 and month 6 (−23.81% and −3.07%, resp.) and was maintained in the alendronate group through month 12 (Figure 3(b)). For the QGY group, the maximal increase in t-P1NP was observed at month 12 (−21.7% versus −10.28% for placebo; *p* < 0.01) and was significantly greater than that observed for month 6 (21.7% versus 11.61%, resp.; *p* < 0.01).

### 3.3. Adverse Events

No significant differences were observed in the overall incidence of clinical adverse events between the two groups (5.0% alendronate versus 7.5% QGY; *p* = 0.513). None of the events were observed with respect to deaths or serious adverse events (Table 2). The incidences of common adverse events were similar between alendronate group and QGY group, such as hypertension (0.0% versus 2.5%, resp.; *p* = 0.155), nausea (3.7% versus 1.2%, resp.; *p* = 0.311), diarrhea (0.0% versus 2.5%, resp.; *p* = 0.155) and fractures (1.2% versus 1.2%, resp.).

## 4. Discussion

This study was initiated to evaluate the superiority of traditional Chinese medicine product, QiangGuYin (QGY), in comparison with the treatment of alendronate, a widely used antiresorptive therapy, and placebo in Chinese postmenopausal osteoporosis by the change of BMD, BTM, and adverse events. To our knowledge, traditional Chinese medicine products are widely used in China and have been a dispute for long; those clinical trials reported online stay in negligible quantity as a result.

Bone, a highly mineralized connective tissue, is continuously broken down and reformed in a process of turnover known as bone remodeling, which occurs through the interaction and balance between bone forming cells called osteoblasts and bone resorbing cells called osteoclasts [13]. The response of *β*-CTX concentrations to antiresorptive therapies has been assessed in clinical studies on postmenopausal women undergoing QGY therapy, as the response of t-P1NP to bone formation therapies accordingly. Researches show that reduction of bone turnover is the main mechanism of how drugs increase BMD and reduce the fracture risk [14]. But the very low levels of BTM could delay the bone microdamage reparation and then affect bone strength. Only one or two indexes cannot represent the overall situations [15, 16]. A new balance between the bone forming and bone resorbing may be necessary and helpful.

Treatment with QGY resulted in significant increase in BMD at the lumbar spine (L1–L4), total hip, trochanter, and femoral neck, with increase in t-P1NP and decrease in *β*-CTX, respectively, compared with placebo. The therapeutic effect was testified by the increasing bone mass and the risk for fractures needs more treatment courses to be proven. To explore the mechanism, compared with the alendronate, the rangeability was wider and the onset time was earlier, but the more interesting finding is that the inhibiting was not continuous as treatment goes on. The new balance may be rebuilt after 9 months of QGY therapy namely. The specific mechanism and length of efficacy duration are the focus of next stage.

In the placebo group, BMD increases in lumbar and decreases in hip. To be honest, this result is common in clinical practice because of the hyperosteogeny of lumbar vertebrae. The total hip BMD may be more reliable in some researches. In the QGY group, 6 cases with bone turnover markers in low level attract attention that 5 of them (t-P1NP, 14.65 ± 3.56 ng/ml, and *β*-CTX, 0.111 ± 0.019 ng/ml) remain BTM steady (*p* > 0.05) in 12 months but 1 case has increased BTM level. Through call visits, patients problems have been solved to different degrees but 3 of them have effective BMD change. Symptoms including knee pain, weakness, aversion to cold, and constipation have been treated actually. The quantity of cases was rare but can verify the efficacy of QGY in postmenopausal women with low bone metabolic rate.

Natural products have modulatory effects on transcription factors, OPG/RANKL system, and signaling pathways (MAPK pathways, BMP pathways, ERs-mediated pathway, oxidative stress-mediated pathways, and NO-mediated pathways) and possess major effects on promoting osteoblasts proliferation and differentiation [17].The composition such as Rhizoma Drynariae [18] upregulates ALP, OPG/RANKL, ER-dependent osteoblastic functions; honeysuckle stem [19] upregulates ALP, Runx2, OCN, OPG/RANKL, and MAPK (MEK/ERK)-mediated ER signaling pathway and possesses effects on promoting osteoblasts proliferation. Total lignans extracted from* Eucommia ulmoides* Oliv. barks inhibited BMD decrease in the femur selected from ovariectomy rat, as was evidenced by the decreased levels of the bone turnover markers, and six compounds derived from them exhibited significant difference in ER subtype (*α* and *β*) which affects the bone metabolism [20, 21]. Simple mix therapeutic effect of Chinese herb which is blindly used to verify the efficacy of compound formulas is one-sided viewpoint.

Bisphosphonates directly inhibit the bone resorption activity through osteoclast and have nothing to do with bone mineralization. Hypocalcemia prohibition and upper gastrointestinal tract stimulation are the major restriction [22]. Compared with other receptor-mediated antiosteoporotic drugs, optional withdrawal will cause the occurrence of withdrawal syndrome which would increase difficulty of future treatment with small probability.

The clinical adverse profiles between the alendronate and QGY groups were similar; most reported adverse events were moderate and under control. TCM related research in clinical settings needs to continue.

## Figures and Tables

**Table 1:** Baseline characteristics. There were no significant differences between treatments based on one-way analysis of variance (ANOVA) for continuous variables.

Characteristic	Alendronate (*N* = 79)	QGY (*N* = 79)	Placebo (*N* = 77)
Age (y)	59.8 ± 4.7	58.8 ± 4.4	59.4 ± 4.5
Years since menopause	11.7 ± 5.5	10.5 ± 5.0	11.6 ± 5.7
Height (cm)	156.2 ± 6.6	156.1 ± 6.7	156.1 ± 6.0
Weight (kg)	56.1 ± 8.8	56.1 ± 8.9	56.2 ± 8.8
BMI	22.8 ± 3.2	23.0 ± 3.5	23.0 ± 3.2
BMD (g/cm^2^); *T*-score [mean (SD)]			
Lumbar spine	0.616 ± 0.049	0.616 ± 0.047	0.615 ± 0.049
	−3.30 ± 0.47	−3.30 ± 0.45	−3.31 ± 0.47
Femoral neck	0.607 ± 0.070	0.606 ± 0.072	0.616 ± 0.064
	−1.91 ± 0.65	−1.92 ± 0.68	−1.83 ± 0.61
Intertrochanter	0.718 ± 0.054	0.720 ± 0.053	0.716 ± 0.053
	−2.23 ± 0.37	−2.22 ± 0.36	−2.25 ± 0.36
Total hip	0.651 ± 0.040	0.650 ± 0.042	0.650 ± 0.042
	−2.11 ± 0.31	−2.11 ± 0.32	−2.10 ± 0.32
Biochemical markers [mean (SE)]			
P1NP (ng/ml)	48.60 ± 2.41	47.14 ± 2.31	48.11 ± 2.44
*β*-CTX (ng/ml)	0.442 ± 0.12	0.437 ± 0.12	0.440 ± 0.12

**Table 2:** Incidence of adverse events.

Event	Alendronate (*N* = 79)	QGY (*N* = 79)
General		
Any adverse events	4 (5.0)	6 (7.5)
Serious	1 (1.2)	2 (2.5)
Death	0 (0.0)	0 (0.0)
Cardiovascular event		
Hypertension	0 (0.0)	2 (2.5)
Discontinuation	0 (0.0)	0 (0.0)
Gastrointestinal event		
Any	3 (3.7)	3 (3.7)
Nausea	3 (3.7)	1 (1.2)
Diarrhea	0 (0.0)	2 (2.5)
Discontinuation	0 (0.0)	1 (1.2)
Musculoskeletal event		
Fractures	1 (1.2)	1 (1.2)
Discontinuation	1 (1.2)	1 (1.2)
